# Impact of red propolis addition on the color stability and surface roughness of glass ionomer cements

**DOI:** 10.2340/biid.v13.45991

**Published:** 2026-05-08

**Authors:** Giovanna Kelly Melo de Lima Sampaio, Pedro Henrique Sette-de-Souza, Basílio Rodrigues Vieira, Gêisa Aiane de Morais Sampaio

**Affiliations:** Department of Dentistry, University of Pernambuco, Arcoverde, Pernambuco, Brazil

**Keywords:** Glass ionomer cements, propolis, roughness, color variation

## Abstract

**Objective:**

The present study aimed to evaluate the surface roughness (SR) and color stability of conventional restorative glass ionomer cements (GICs) modified with the addition of red propolis ethanolic extract (RPEE) as an antimicrobial agent.

**Methodology:**

Four GICs (Riva, Maxxion, Vidrion, and Ketac) were used with the addition of RPEE at concentrations of 11% and 20%. For the control groups, the GICs were manipulated according to the manufacturers’ instructions. SR was assessed using a surface profilometer. For the color variation analysis, the specimens’ colors were measured in the CIE L*a*b* color space using a spectrophotometer. The Kruskal–Wallis test, followed by Dunn’s test, was used to evaluate statistical significance.

**Results:**

The addition of RPEE did not negatively affect the SR of Riva, Maxxion, and Ketac GICs (*p* = 0.796, *p* = 0.111, and *p* = 0.858, respectively). By contrast, a significant reduction in SR was observed in Vidrion GIC with the addition of RPEE (*p* = 0.009). When comparing the different GIC brands, Riva and Ketac exhibited the lowest SR, with significant differences relative to Vidrion (*p* = 0.008) and Maxxion (*p* = 0.006). Both RPEE concentrations (11% and 20%) caused major color changes in all GICs tested, with no statistical differences between the two concentrations (*p* = 1.000), nor among the different GIC brands (*p* = 0.071).

**Conclusion:**

The incorporation of RPEE into conventional GIC did not increase SR, though in the case of Vidrion cement, it resulted in a significant reduction of this parameter. Conversely, the addition of red propolis at both 11% and 20% concentrations caused significant and clinically unacceptable color changes in all tested cements, regardless of brand or concentration.

## Introduction

Glass ionomer cements (GICs) constitute a class of materials widely used in dental practice due to their properties of continuous fluoride release, biocompatibility with oral tissues, and chemical bonding to dental tissues. These characteristics make them particularly suitable for atraumatic restorative treatments, minimally invasive procedures, and clinical situations involving patients with a high risk of caries [1, 2]. The incorporation of antimicrobial agents into restorative materials has been investigated as a promising strategy to enhance their clinical performance by reducing microbial activity at the tooth–restoration interface and minimizing the risk of secondary failures [3–7].

Propolis is a material produced by bees from resins collected from plant stems. Interest in its therapeutic use has increased considerably following scientific evidence supporting its role in disease prevention due to its various biological properties, including antimicrobial, anti-inflammatory, antioxidant, antiviral, and wound-healing activities [8–10].

Red propolis is found in the Northern and Northeastern regions of Brazil, particularly in coastal areas, and is distinguished by its well-recognized antibacterial, anti-inflammatory, and antioxidant potential, attributed mainly to its high content of flavonoids and phenolic compounds [11–13]. Due to its antibacterial activity against oral microorganisms such as *Streptococcus mutans*, the incorporation of red propolis ethanolic extract (RPEE) into GICs has been shown to enhance their antimicrobial potential. Thus, its use as an additive in GICs emerges as an appealing alternative in restorative dentistry [6, 14–16].

Nevertheless, any modification in the composition of these materials must preserve their essential physical and mechanical properties [17], such as surface roughness (SR) – directly associated with biofilm retention and wear – and color stability, an indispensable parameter for esthetic restorations [18, 19]. Significant alterations in these characteristics may restrict the clinical applicability of the material, even in the presence of potential antimicrobial benefits. Given this context, it becomes relevant to investigate whether the addition of RPEE to GICs compromises or alters their surface and optical properties. Therefore, the present study aimed to evaluate the effects of incorporating RPEE, at different concentrations, on the SR and color stability of various brands of restorative GICs. The null hypothesis is that the incorporation of RPEE does not alter the SR and color stability of cements.

## Materials and methods

The analyses were conducted at the Multiuser Laboratory of Biomaterials from the Caatinga (BIOMA), University of Pernambuco.

### Preparation of GICs containing RPEE

The present study used RPEE at concentrations of 11% and 20% sourced from the coastal region of Alagoas, Brazil (Fernão Velho, Alagoas, Brazil, Batch 03/23). The concentrations of 11% and 20% were selected based on previous studies investigating antimicrobial and biological properties of red propolis-modified GICs [6, 15, 16]. Four restorative GICs were used: Riva (SDI, Victoria, Australia), Maxxion R (FGM, Joinville, Brazil), Vidrion (SSWhite, Rio de Janeiro, Brazil), and Ketac Molar Easymix (3M, USA) (Table 1).

**Table 1:** Manufacturer, composition, and batch of the GICs tested.

GIC	Manufacturer	Liquid	Powder	Batch
Riva	SDI (Australia)	Polyacrylic acid, dihydroxybutanedioic acid, water	Chemical glass oxides, polyacrylic acid	11875203
Maxxion	FGM (Brazil)	Polyacrylic acid, water	Fluorine-aluminum silicate glass, tartaric acid, calcium fluoride	181022
Vidrion	SSWHITE (Brazil)	Tartaric acid, water	Sodium-calcium-aluminum fluorosilicate, barium sulfate, polyacrylic acid, pigments	W015157
Ketac Molar Easymix	3M ESPE (Germany)	Copolymer of acrylic acid and maleic acid, tartaric acid, water	Chemical glass oxides, polyacrylic acid	10006917

For the control groups, the cements were manipulated according to the manufacturers’ instructions. In the experimental groups, RPEE solutions at concentrations of 11% and 20% were incorporated into the cement liquid during manipulation, using a 1:1 ratio (one drop of tartaric acid-based liquid to one drop of RPEE solution), measured with the same dispensing tip, and subsequently spatulated with the powder. The total liquid volume recommended by the manufacturer was maintained; however, 50% of the original liquid was replaced by RPEE (1:1 liquid:RPEE ratio), ensuring that the overall liquid-to-powder proportion remained unchanged.

The materials were placed in polyethylene molds (5 mm × 2 mm), with the surface covered by a glass slide, and allowed to set for 5 min at 25 °C. A total of 60 samples (*n* = 5) were fabricated for each test and distributed across 12 groups: Group RC (Riva Control), Group R11 (Riva with 11% RPEE), Group R20 (Riva with 20% RPEE), Group MC (Maxxion Control), Group M11 (Maxxion with 11% RPEE), Group M20 (Maxxion with 20% RPEE), Group VC (Vidrion Control), Group V11 (Vidrion with 11% RPEE), Group V20 (Vidrion with 20% RPEE), Group KC (Ketac Control), Group K11 (Ketac with 11% RPEE), and Group K20 (Ketac with 20% RPEE). After manipulation, all specimens were stored at 37°C under 100% relative humidity for 24 h prior to SR and color analyses, ensuring completion of the initial acid–base setting reaction.

For the SR (Ra) evaluation test, a surface profilometer (Surftest 301 J, Mitutoyo, Kanagawa, Japan) was used at a speed of 0.25 mm/s with a cutoff value of 0.8 mm. The mean value of three readings was calculated and used for subsequent statistical analysis.

For the color variation analysis, the specimen colors were measured in the CIE L*a*b* color scale using a spectrophotometer (Vita Easyshade Advance, Vita Zahnfabrik, Bad Säckingen, Germany). The specimens were thoroughly dried with a paper towel and positioned in direct contact with the spectrophotometer’s measuring probe. The device was calibrated on the standard white reference according to the manufacturer’s instructions. Three repeated measurements were obtained for each specimen, and the color values were determined by calculating the mean of the recorded data. The control group data were treated as the baseline color (L0*, a0*, b0*) and compared with each test group (L1*, a1*, b1*). The differences between baseline and final color values (ΔE*) were calculated according to the following formula: ΔE* = [(L1* – L0*)² + (a1* – a0*)² + (b1* – b0*)²]½.

The results obtained were organized into a database using Microsoft Excel and subsequently exported to the Statistical Package for the Social Sciences (version 20, SPSS, Inc., Chicago, IL, USA). The Kolmogorov–Smirnov test was used to assess data normality. The nonparametric Kruskal–Wallis test, followed by Dunn’s multiple comparison test, was applied to determine statistical significance. All tests were conducted based on a 95% confidence level (*p* < 0.05).

## Results

Regarding the SR data of the GICs, when comparing the cements with added RPEE to their respective controls, it was observed that the addition of RPEE did not negatively affect the SR of Riva, Maxxion, and Ketac cements (*p* = 0.796, *p* = 0.111, and *p* = 0.858, respectively). However, for Vidrion GIC, a significant reduction in SR was observed in the groups with added RPEE (*p* = 0.009), thereby improving its surface smoothness (Table 2).

**Table 2:** Mean and standard deviation of roughness (Ra) values, comparison between control and RPEE groups, and comparison among different GIC brands.

	Riva	Maxxion	Vidrion	Ketac	*p* *
**Control**	0.57 (0.30)^a^	1.18 (0.40)^ab^	1.32 (0.28)^Ab^	0.58 (0.30)^a^	**0.008**
**RPEE 11%**	0.56 (0.09)^a^	1.34 (0.65)^b^	0.81 (0.17)^Bab^	0.54 (0.14)^a^	**0.006**
**RPEE 20%**	0.60 (0.13)^a^	0.83 (0.22)^ab^	0.89 (0.06)^Bb^	0.60 (0.13)^a^	**0.008**
*p* *	0.796	0.111	**0.009**	0.858	–

* Means followed by the same letters do not represent statistically significant differences (*p* > 0.05). Means followed by different letters represent statistically significant differences (*p* < 0.05).

A,a Capital letters express differences between the control and test groups in the same cement (in a column); lowercase letters express the comparison between the cements (in a row).

Kruskal–Wallis nonparametric test followed by Dunn’s multiple comparison test (p < 0.05).

Regarding the comparison of SR among different GIC brands, it was observed that, in the control groups, Riva and Ketac cements exhibited the lowest SR, with significant differences compared with Vidrion (*p* = 0.008). For the groups containing 11% RPEE, Riva and Ketac also demonstrated the lowest roughness values, with a significant difference in comparison to Maxxion (*p* = 0.006). For the groups containing 20% RPEE, Riva and Ketac continued to present the lowest SR values, showing significant differences relative to Vidrion (*p* = 0.008) (Table 2).

Regarding the color variation data of the GICs after the addition of RPEE, both concentrations – 11% and 20% – caused substantial color changes in all GICs tested, with no statistically significant differences between the two concentrations (*p* = 1.000), nor among the different cement brands (*p* = 0.071) (Table 3).

**Table 3:** Color variation between control and test groups, comparison between RPEE concentrations (ΔE), and comparison among different GIC brands (ΔE).

	Riva	Maxxion	Vidrion	Ketac	*p* *
**RPEE 11%**	96.8	71.9	113.2	120	0.071
**RPEE 20%**	96.8	71.9	113.2	120	0.071
*p* *	1.000	1.000	1.000	1.000	–

* Means followed by the same letters do not represent statistically significant differences (*p* > 0.05). Means followed by different letters represent statistically significant differences (*p* < 0.05).

A,aCapital letters express differences between the control and test groups in the same cement (in a column); lowercase letters express the comparison between the cements (in a row).

Kruskal–Wallis nonparametric test followed by Dunn’s multiple comparison test.

## Discussion

The results of the present study demonstrated that the addition of RPEE, at concentrations of 11% and 20%, did not promote a significant increase in SR for most of the evaluated GICs. These findings are in agreement with previous studies reporting that the incorporation of natural or antimicrobial agents does not necessarily compromise the surface topography of GICs when used at appropriate concentrations [20, 21]. The maintenance of low SR values is clinically relevant, as smoother surfaces are associated with reduced biofilm retention, lower susceptibility to extrinsic staining, and increased longevity of restorations [22, 23].

A significant reduction in SR was observed for the Vidrion GIC after the addition of RPEE. This finding may be related to differences in chemical composition and glass particle size distribution among commercial GIC brands, factors that directly influence the acid–base reaction, the formation of the polysalt matrix, and interactions with liquid additives [24]. It is possible that RPEE acted as a matrix modifier in this specific cement, promoting better particle accommodation or filling of surface micro-irregularities. Previous studies have reported that certain additives may exert a plasticizing effect or modify the viscosity of the mixture, positively influencing surface smoothness after setting [25, 26].

The comparison among different GIC brands showed that Riva and Ketac exhibited lower SR values than Vidrion and Maxxion, regardless of RPEE addition. These results are consistent with the literature, which highlights significant variations in the physicomechanical properties of GICs depending on formulation differences, such as powder-to-liquid ratio, type of polyacrylic acid, presence of modifiers, and quality of the fluoroaluminosilicate glass [27–29]. Therefore, the choice of restorative material remains a decisive factor for clinical performance, even when antimicrobial modification strategies are employed. The variability observed among different GIC brands reinforces that compositional differences – such as glass particle size, type of polyacid, and presence of modifiers – directly influence the interaction with additives. Therefore, the present findings cannot be universally extrapolated to all glass ionomer formulations without specific testing.

In contrast, color stability was significantly affected by the addition of RPEE in all tested GICs, with ΔE values considered clinically unacceptable according to widely accepted thresholds in the literature (ΔE > 3.3 or > 2.7, depending on the adopted criterion) [30, 31]. The intense pigmentation characteristic of red propolis, associated with the presence of chromophoric phenolic compounds, explains the pronounced color changes observed, regardless of cement brand or extract concentration. The final appearance of the specimens was visibly reddish, which is consistent with the extremely high ΔE values (ranging from 71.9 to 120) and clinically incompatible with esthetic restorative demands. Similar findings have been reported in studies incorporating propolis or other pigmented natural extracts into restorative materials, highlighting a recurring challenge in maintaining dental esthetics [32, 33].

The absence of statistically significant differences between the 11% and 20% concentrations suggests that even lower levels of RPEE are sufficient to induce marked color alterations, limiting its use in esthetically demanding areas. However, this effect may be clinically acceptable for provisional restorations, posterior regions, or specific therapeutic applications in which antimicrobial benefits outweigh esthetic requirements [34, 35]. Alternatively, strategies such as extract encapsulation, purification of less pigmented fractions, or incorporation of RPEE into inner layers of the restorative material may be explored to minimize visual impact [36].

Overall, the findings of the present study indicate that the addition of RPEE is promising in terms of maintaining SR of GICs, without compromising – and in some cases even improving – this property. Nevertheless, the significant color changes represent an important limitation for its clinical application in definitive esthetic restorations. Although smoother surfaces are associated with reduced plaque accumulation, SR should not be interpreted in isolation. The partial substitution of the original liquid by RPEE may influence the acid–base reaction and potentially affect mechanical strength and wear resistance. Therefore, improved smoothness does not necessarily indicate enhanced clinical performance without complementary mechanical testing.

Limitations of the present study include its in vitro design, the short evaluation period (24 h), the absence of mechanical strength testing, and the lack of artificial aging protocols. These factors limit direct prediction of long-term clinical performance. Further investigations should assess the long-term behavior of these modified materials, including mechanical strength, fluoride release and recharge capacity, sustained antimicrobial activity, and artificial aging, in order to establish safer and more effective protocols for the use of red propolis as an additive in GICs.

## Conclusion

The incorporation of RPEE into conventional GIC did not increase SR and, in the case of the Vidrion cement, resulted in a significant reduction of this parameter. Conversely, the addition of red propolis at both 11% and 20% concentrations caused significant and clinically unacceptable color changes in all tested cements, regardless of brand or concentration. These findings suggest that while red propolis may be a viable antimicrobial additive from a surface integrity standpoint, its negative impact on color stability limits its use in esthetically critical restorative applications.
